# Clinical and laboratory findings in 503 cattle with traumatic reticuloperitonitis

**DOI:** 10.1186/s12917-018-1394-3

**Published:** 2018-03-05

**Authors:** Ueli Braun, Sonja Warislohner, Paul Torgerson, Karl Nuss, Christian Gerspach

**Affiliations:** 1 Department of Farm Animals, Vetsuisse-Faculty, University of Zurich, Winterthurerstrasse 260, CH-8057 Zurich, Switzerland; 2 Section of Epidemiology, Vetsuisse-Faculty, University of Zurich, Winterthurerstrasse 260, CH-8057 Zurich, Switzerland

**Keywords:** Cattle, Traumatic reticuloperitonitis, Foreign body tests, Haematological findings, Biochemical findings

## Abstract

**Background:**

The study evaluated the results of clinical examination and haematological and serum biochemical analyses in 503 cattle with traumatic reticuloperitonitis (TRP).

**Results:**

The most common clinical findings were abnormal demeanour and general condition (87%), decreased rumen motility (72%), poorly digested faeces (57%), decreased rumen fill (49%), fever (43%) and tachycardia (26%). In 58% of the cattle, at least one of three tests for reticular foreign bodies (pinching of the withers, pressure on the xiphoid and percussion of the abdominal wall) was positive, and in 42% all three tests were negative. The most common haematological findings were decreased haematocrit in 45% of cattle and leukocytosis in 42%. An increase in the concentration of fibrinogen in 69% of cattle and total protein in 64% were the main biochemical findings. The glutaraldehyde test time was decreased with coagulation occurring within 6 min in 75% of cattle.

**Conclusions:**

In many cases, a diagnosis of TRP is not possible based on individual clinical or laboratory findings because even the most common abnormalities are not seen in all cattle with TRP.

## Background

Traumatic reticuloperitonitis (TRP) remains one of the most important internal disorders of cattle in addition to abomasal displacement. One of the first large studies found that the incidence of TRP was as high as 80% [1], but more recent reports have shown that it is now approximately 2–12% [2–5]. Despite this decrease, the clinical implications remain the same. Traumatic reticuloperitonitis most commonly results from perforation of the reticulum by metal objects such as nails or wire that have been accidently incorporated into feed and ingested [6–9]. This may lead to localised peritonitis, sometimes involving neighbouring organs, and in severe cases it results in generalised peritonitis. Clinical signs of TRP have been described in a number of reference texts [6, 8, 9]. Acute disease usually results in distinct signs that include anorexia, decreased milk production, fever, ruminal atony and tympany, abdominal pain, arched back, abdominal guarding and tense abdomen [6, 8, 9]. The clinical signs in cattle with chronic disease on the other hand are often less apparent. A short audible grunt is considered characteristic of acute TRP and may be a spontaneous response to reticular contractions or changes in posture such as lying down and getting up [6, 10, 11]. There may be additional signs in cattle with sequelae such as traumatic pericarditis [12], liver abscesses [13] or cranial functional stenosis [6, 9]. Cattle with acute localised peritonitis typically have neutrophilia with a regenerative left shift [9] and those with acute diffuse peritonitis have leukopenia with a degenerative left shift [9]. The most important biochemical findings are increased concentrations of total protein and fibrinogen. Tests for reticular foreign bodies may elicit a grunt, although other painful disorders of the thorax and abdomen may stimulate the same reaction [6, 10, 14]. Pinching of the withers, gradual application of pressure followed by sudden release of pressure on the area between the xiphoid and the umbilicus using a pole and percussion of the abdominal wall with a rubber hammer over the region of the reticulum are the most useful foreign body tests for TRP [15]. Others include the zone test developed by Kalchschmidt, leading the animal up and down a steep incline and ferroscopy to detect the presence of metal [15]. Pinching of the withers, abdominal percussion and the pole test have been the clinical tests used in more than 20,000 cattle over more than 30 years in our clinic. Other diagnostic tests include radiography and ultrasonography, which will be addressed in a separate paper. The clinical and laboratory findings in cattle with TRP have been thoroughly described in a number of standard texts. However, the majority of information is largely based on empirical evidence, and systematic evaluation of the clinical and laboratory findings in cattle with a definitive diagnosis of TRP has not yet been done. In particular, the frequency of positive responses to foreign body tests, which are considered an essential part of a diagnostic work-up in cattle with TRP, has not been determined. The goals of the present study were to describe the clinical and laboratory findings in 503 cattle with TRP, to establish the frequency of positive responses to foreign body tests and to determine which foreign body test elicited the most positive responses.

## Methods

### Animals

This was a retrospective study of 503 cattle that had a main diagnosis of TRP, which meant that the clinical signs were attributable to TRP and not another concomitant disease or disorder. The cattle were all greater than 1 year of age and had been admitted to the Veterinary Teaching Hospital, University of Zurich, from January 1, 2001 to December 31, 2014. The diagnosis of TRP was based on the results of ultrasonography, radiography, laparoruminotomy and/or postmortem examination. Cattle with TRP that had concomitant diseases causing anterior abdominal or caudal thoracic pain were excluded; this included 27 cows with bronchopneumonia and 24 cows with abomasal ulcers. All cattle that had been part of previous reports were not included in the present study. Traumatic reticuloperitonitis was diagnosed based on radiographic evidence of a foreign body that penetrated or perforated the reticular wall or was seen outside of the reticulum in 225 cattle and on ultrasonographic changes of the reticular wall in 403 cattle. Foreign bodies that penetrated or perforated the reticular wall were removed during laparoruminotomy in 196 cattle, and in 10 others, a reticular abscess was drained transcutaneously under ultrasonographic guidance. In all 61 cattle that were euthanased because of a poor prognosis, TRP was confirmed during postmortem examination. In all cattle, the diagnosis of TRP was based on more than one criterion. The results of ultrasonography, radiography, surgical treatment and postmortem examination as well as the outcome of treatment were described in a dissertation [16]. There were 496 females and 7 males, which ranged in age from 1.0 to 14.9 years (median, 4.1 years) with 97% of the cattle being more than 2 years of age. Breeds included Swiss Braunvieh (208), Holstein-Friesian (155), Simmental (124), Jersey (3), Eringer (1), Hinterwälder (1) and crossbred cattle (11). The length of illness ranged from 1 to 90 days (median, 4 days). The majority of cows (*n* = 168, 33%) had calved 0 to 8 weeks before becoming ill; this incidence was significantly higher than that of other reproductive stages (*P* < 0.01). There were no significant differences between the other reproductive stages. Of the 503 cattle, 58 had received no treatment before referral, 50 had been treated with an antibiotic, 88 had received a magnet and 209 had received an antibiotic and a magnet. A non-steroidal anti-inflammatory drug or metamizole was used in addition to other treatments in 183 cattle or exclusively in 11 cattle.

### Clinical examination

The cattle underwent a thorough clinical examination [17]. The general health condition was evaluated by determining demeanour, appearance of hair coat and muzzle, skin elasticity, position of the eyes in relation to the sockets and skin surface temperature. Each animal was observed for signs of pain such as spontaneous grunting and bruxism. The general health condition was considered to be mildly to moderately abnormal when appetite and degree of alertness were decreased and severely abnormal when there was anorexia and apathy or constant bruxism or grunting. The rumen was assessed for degree of fill, number and intensity of contractions and layering of contents. Sensitivity in the reticular region was assessed by preventing the animal from breathing for a short period by placing a plastic rectal sleeve over the mouth and nose and listening for grunting during the following deep breath. This was followed by the foreign body tests, which included the pole test, pinching of the withers and percussion of the abdominal wall over the region of the reticulum with a rubber hammer. Each test was carried out four times, and the reaction of the animal was observed each time. A test was considered positive when it elicited a short grunt three out of four times. The response to a test was considered questionable when it elicited a grunt two out of four times and negative when the animal did not grunt or grunted only once. Swinging and percussion auscultation as well as a rectal examination were also carried out. Faeces were assessed for colour, consistency, amount, fibre particle length and abnormal contents.

### Urinalysis

In 445 cattle, a urine sample was collected during spontaneous micturition, but in 33 cases catheterisation of the bladder was carried out. The colour and transparency of the urine were assessed macroscopically, and the specific gravity was determined using a refractometer (HRMT 18, A. Krüss Optronic GmbH, Hamburg, Germany). A urine test strip (Combur9^®^, Roche, Basel) was used to determine urine pH and the presence of protein, erythrocytes, glucose, ketones, leukocytes, nitrite, urobilinogen and bilirubin.

### Rumen fluid analysis

A sample of rumen fluid (200 to 300 ml) was collected using a Dirksen probe [15] and assessed for colour, odour, consistency and pH. In addition, a methylene blue reduction time and the concentration of chloride were determined. The concentration of chloride in rumen fluid was carried out using an MK-II-Chloride Analyser 9265 (Sherwood, Cambridge).

### Haematological and serum biochemical analyses

The following blood samples were collected from all cattle: 5 ml of EDTA blood for haematological analysis, 10 ml of whole blood for serum biochemistry, 2 ml of whole blood mixed with 0.2 ml heparin for venous blood gas analysis and 5 ml of EDTA blood for the glutaraldehyde test. Haematological analysis included the determination of PCV, total leukocyte count and the concentrations of fibrinogen and total protein using an automated blood analyzer (CELL-Dyn 3500, Abbott Diagnostics Division, Baar). A differential leukocyte count was done in cattle with leukopenia (< 5000 leukocytes/μl blood) or leukocytosis (> 10,000 leukocytes/μl blood). The concentrations of serum urea nitrogen and bilirubin and the activities of the enzymes aspartate aminotransferase (ASAT), γ-glutamyltransferase (γ-GT) and glutamate dehydrogenase (GLDH) were determined at 37 °C using an automated analyser (Cobas-Integra-800-Analyser, Roche Diagnostics, Basel) and the manufacturer’s reagents (Roche-Reagents) according to the International Federation of Clinical Chemistry and Laboratory Medicine (IFCC). Venous blood gas analysis was done using an automated analyser (RapidLab 248, (Siemens Schweiz AG, Zurich). A glutaraldehyde test (Glutaltest^®^, Graeub AG, Bern) was performed according to the manufacturer’s instructions. Results were compared to reference intervals recently reported [18].

### Statistical analysis

The program IBM SPSS Statistics 22.0 was used for analysis. Frequencies were determined for each clinical and laboratory variable. The Wilk-Shapiro test was used to test the data for normality. Means ± standard deviations were calculated for normal data (rectal temperature, urine-pH, urine specific gravity, lymphocyte count, fibrinogen concentration and venous blood pH) and medians for non-normal data (heart rate, respiratory rate, rumen pH, rumen chloride concentration, haematocrit, white blood cell, neutrophil count, total protein, urea nitrogen and bilirubin concentrations, ASAT, γ-GT and GLDH activities, glutaraldehyde test time and pCO_2_, HCO_3_^−^ and base excess of venous blood). Differences in seasonal incidence of TRP and differences in occurrence at various reproductive stages were analysed using a one-way analysis of variance and the post hoc Bonferroni test. The 3-month periods of January to March, April to June, July to September and October to December, and the reproductive stages, which included the first 8 weeks postpartum, > 8 weeks postpartum and open, and 3 months, 4 to 6 months and 7 to 9.5 months of gestation were compared. A value of *P* < 0.05 was considered significant.

## Results

Over the 14-year-study period, cattle with TRP constituted a yearly average of 7.1% (range, 5.1–9.1%) of the bovine patients treated for internal disorders.

### Seasonality

**Fig. 1:**
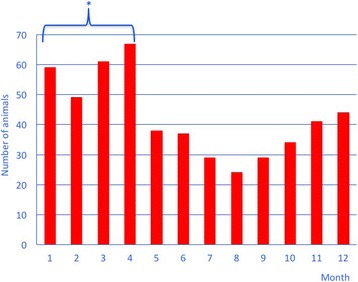
Seasonal occurrence of traumatic reticuloperitonitis in cattle over a period of 14 years. * Difference to May to August and to September to December *P* < 0.05

There were significantly more cattle (*n* = 232, 46%) with TRP seen in the months of January to April than May to August (*n* = 146, 29%) or September to December (*n* = 125, 25%) (*P* < 0.01) (Fig. 1).

### Clinical findings

The general demeanour was normal in 65 (13%) cattle, mildly to moderately abnormal in 425 (84%) and markedly abnormal in 13 (3%). The rectal temperature varied from 36.4 to 41.3 °C (39.0 ± 0.7 °C) (Table 1) and was mildly to severely increased (39.1–41.3 °C) in 217 (43%) cattle. The heart rate ranged from 40 to 162 bpm (median, 76 bpm), and bradycardia was found in 24 (5%) cattle and tachycardia in 130 (26%). The respiratory rate was 12 to 100 breaths per minute (median, 28 breaths per min), and 102 (21%) cattle had bradypnoea and 42 (8%) had tachypnoea. The type of respiration was costoabdominal in 86% of cattle, costal in 1% and abdominal in 13%.

**Table 1:** Rectal temperature and heart and respiratory rates in cattle with traumatic reticuloperitonitis

Variable	Finding	Range	Number of cattle	Percent
Rectal temperature (39.0 ± 0.7 °C) (*n* = 503)	Normal	38.0–39.0	263	52
	Decreased	36.4–37.9	23	5
	Mildly increased	39.1–39.5	146	29
	Moderately increased	39.6–40.0	45	9
	Severely increased	40.1–41.3	26	5
Heart rate (median = 76 bpm) (*n* = 502)	Normal	60–80	348	69
	Decreased	40–59	24	5
	Mildly increased	81–100	108	22
	Moderately increased	101–120	15	3
	Severely increased	121–162	7	1
Respiratory rate (median = 28 breaths per min) (*n* = 501)	Normal	21–40	357	71
	Decreased	12–20	102	21
	Increased	41–100	42	8

Rumen fill was decreased in 242 (49%) cattle, and in 70 (14%) the rumen appeared fuller than normal (Table 2). Rumen motility was decreased in 354 (72%) cattle, and the layering of rumen contents was abnormal or absent in 227 (45%). Ruminal tympany was present in 51 (10%) cattle in addition to other ruminal abnormalities.

**Table 2:** Results of rumen evaluation in cattle with traumatic reticuloperitonitis

Variable	Finding	Number of cattle	Percent
Rumen fill (*n* = 495)	Normal	183	37
	Reduced	242	49
	Fuller than normal	70	14
Rumen motility (*n* = 489)	Normal	135	28
	Reduced	325	66
	Absent	29	6
Ruminal contractions per 2 min. (*n* = 502)	Normal (2–3)	268	53
	None	146	29
	1	72	14
	4 to 6	16	3
Stratification (*n* = 499)	Normal	185	37
	Reduced	195	39
	Absent	32	6
	Tympanic	51	10
	Firm content	36	7

Signs of pain occurred spontaneously in 179 (36%) cattle and included arching of the back (*n* = 68), bruxism (*n* = 80) and grunting (*n* = 8). Twenty-three cattle had both arching of the back and bruxism. Walking on a lead rope elicited grunting in another five cattle. Bruxism was elicited in 43 (9%) cattle, and at least one grunt, rarely more, was heard in 20 (4%) after temporary interruption of breathing using a plastic rectal sleeve over the mouth and nose. Of the foreign body tests, a positive response was seen with the pole test in 210 (43%), pinching of the withers in 191 (39%) and pain percussion in 120 (24%); this meant that the tests elicited grunting a minimum of three of the four times the test was done (Table 3). A positive response was seen with one of the three tests in 57 (19%) cattle, with two of the tests in 52 (17%) and with all three tests in 66 (22%). Thus, a minimum of one of the tests was positive in 175 (58%) cattle, and all three tests were negative in 129 (42%).

**Table 3:** Results of foreign body tests in cattle with traumatic reticuloperitonitis

Test	Finding	Number of cattle	Percent
Pole test (*n* = 488)	Positive	210	43
	Questionable	72	15
	Negative	206	42
Pinching of the withers (*n* = 495)	Positive	191	39
	Questionable	80	16
	Negative	224	45
Pain percussion (*n* = 494)	Positive	120	24
	Questionable	91	19
	Negative	283	57

Positive: at least three of four tests elicited a grunt

Questionable: two of four tests elicited a grunt

Negative: none or one of four tests elicited a grunt

Swinging and percussion auscultation on the left side of the animal was positive in 26 (5%) cattle. The same test on the right side of the animal was positive in 87 (17%) cattle.

Intestinal motility was decreased or absent in 252 (50%) cattle. Faecal consistency was watery to loose in 121 (30%), abnormally thick in 70 (14%) and partly liquid and partly firm in 16 (3%). The degree of comminution of faeces based on fibre particle length was abnormal in 286 (57%), and the amount of faeces produced was decreased in 177 (35%).

Rectal examination revealed distension of the rumen in 98 (19%) cattle. The rumen felt firm in 51 (10%) and tympanic in 25 (5%) and was L-shaped in 15 (3%). There was loss of negative pressure in 21 (4%) cattle and crepitus in 8.

### Urinalysis

Urine pH was higher than normal in 203 (42%) cattle, and the urine specific gravity was lower than normal in 212 (46%) (Table 4). Proteinuria was found in 57% and haematuria in 72% of cattle; however, in most cases these abnormalities were mild and likely due to contamination. A few cattle had glucosuria (11%) or ketonuria (9%).

**Table 4:** Results of urinalysis in cattle with traumatic reticuloperitonitis

Variable Mean ± sd	Finding	Number of cattle	Percent
Transparency (*n* = 474)	Transparent	463	98
	Opaque	11	2
pH (*n* = 478) 7.9 ± 1.15	Normal (7.0–8.0)	203	42
	Decreased (5.0–6.9)	73	16
	Increased (8.1–9.0)	203	42
Specific gravity (*n* = 461) 1020.6 ± 11.18 g/l	Normal (1020–1040)	229	50
	Decreased (1000–1019)	212	46
	Increased (1021–1060)	20	4
Protein concentration (*n* = 478)	Negative (< 30 mg/dl)	204	43
	+ (approx. 30 mg/dl)	251	53
	++ (approx. 100 mg/dl)	21	4
Erythrocytes (*n* = 478)	Negative	348	73
	+ (approx. 5–10)	61	13
	++ (approx. 25)	29	6
	+++ (approx. 50)	40	8
Glucose concentration (*n* = 478)	Negative (< 50 mg/dl)	425	89
	+ (approx. 50 mg/dl)	24	5
	++ (approx. 100 mg/dl)	18	4
	+++ (≥ 300 mg/dl)	12	2
Ketone bodies (*n* = 477)	Negative (< 10 mg/dl)	438	91
	+ (approx. 10 mg/dl)	19	4
	++ (approx. 50 mg/dl)	12	3
	+++ (≥ 150 mg/dl)	8	2

+ Mildly increased

++ Moderately increased

+++ Severely increased

### Rumen fluid analysis

The pH of rumen fluid was greater than 7.0 in 312 (72%) cattle (Table 5). Based on the results of the methylene blue reduction test, rumen fluid was considered inactive in 236 (73%) cattle. The concentration of chloride was decreased in 88 (20%) cattle and increased in 113 (26%) compared with the reference interval.

**Table 5:** Results of rumen fluid analysis in cattle with traumatic reticuloperitonitis

Variable	Finding	Number of cattle	Percent
pH (*n* = 432) median = 8	Normal (5.5–7.0)	118	28
	Increased (7.1–10.0)	312	72
Methylene blue reduction (min.) (*n* = 323)	Hyperreactive (< 3 Min.)	19	6
	Moderately reactive (3–6 Min.)	68	21
	Inactive (> 6 Min.)	236	73
Chloride concentration (*n* = 435) median = 20 mmol/l	Normal (15–25)	234	54
	Decreased (6–14)	88	20
	Increased (26–99)	113	26

### Haematological and serum biochemical analyses

The most common abnormalities in blood analysis were a decrease in haematocrit in 224 (45%) cattle and leukocytosis in 209 (42%) (Table 6). Neutrophilia occured in 90% of animals with leukocytosis and lymphopenia was noted in 36%. The most common abnormalities in the biochemical profile were an increase in the concentration of fibrinogen in 345 (69%) and total protein in 319 (64%) cattle. The coagulation time in the glutaraldehyde test was less than 6 min in 371 (75%) cattle. Venous blood pH was lower than normal in 170 (36%) cattle and higher than normal in 88 (18%) (Table 7).

**Table 6:** Haematological and blood biochemical findings in cattle with traumatic reticuloperitonitis

Variable (mean ± sd or median)	Finding	Number of cattle	Percent
Haematocrit (%) (*n* = 501) median = 30%	Normal (30–35)	218	43
	Decreased (18–29)	224	45
	Increased (36–60)	59	12
White blood cell count (/μl) (*n* = 501) median = 9400/μl	Normal (5000–10,000)	272	54
	Decreased (1500–4999)	20	4
	Increased (10,001–29,200)	209	42
Neutrophil count (/μl) (*n* = 211) median = 8568/μl	Normal (1230–3350)	17	8
	Decreased (380–1229)	4	2
	Increased (3351–26,280)	190	90
Lymphocyte count (/μl) (*n* = 222) 2576 ± 1016/μl	Normal (2190–5120)	137	62
	Decreased(150–2189)	81	36
	Increased (5121–7040)	4	2
Fibrinogen concentration (*n* = 499) (8.6 ± 3.1 g/l)	Normal (4–7)	131	26
	Decreased (2.0–3.9)	23	5
	Increased (7.1–17.0)	345	69
Total protein concentration (*n* = 501) (median = 84 g/l)	Normal (60–80)	177	35
	Decreased (45–59)	5	1
	Increased (81–122)	319	64
Urea concentration (*n* = 501) (median = 4.0 mmol/l)	Normal (2.4–6.5)	373	74
	Decreased (0.7–2.3)	58	12
	Increased (6.6–33.5)	70	14
Bilirubin concentration (*n* = 500) (Median = 4.6 μmol/l)	Normal (≤ 6.5)	359	72
	Increased (6.6–71.5)	141	28
ASAT activity (*n* = 501) (median = 69.0 U/l)	Normal (≤ 103)	425	85
	Increased (104–812)	76	15
γ-GT activity (*n* = 500) (median = 24.0 U/l)	Normal (≤ 30)	407	81
	Increased (31–154)	93	19
GLDH activity (*n* = 191) (median = 15.0 U/l)	Normal (≤ 25.0)	127	67
	Increased (25.1–522.0)	64	33
Glutaraldehyde test (*n* = 498) median = 3.5 min.	Normal (≥ 10 min.)	91	18
	6.1–9.9 min.	36	7
	3.1–6.0 min.	128	26
	≤ 3 min.	243	49

*ASAT* Aspartate aminotranferase, *γ-GT* γ-glutamyltransferase, *GLDH* glutamate dehydrogenase

**Table 7:** Venous blood gas analysis in cattle with traumatic reticuloperitonitis

Variable (median; mean ± sd)	Finding	Number of cattle	Percent
pH (*n* = 474) 7.42 ± 0.05	Normal (7.41–7.45)	216	46
	Decreased (7.20–7.40)	170	36
	Increased (7.46–7.58)	88	18
pCO_2_ (*n* = 472) (median = 43.3 mmHg)	Normal (35–45)	254	54
	Decreased (24.1–34.9)	45	9
	Increased (45.1–75.6)	173	37
HCO_3_^−^ (*n* = 474) (median = 26.5 mmol/l)	Normal (20.0–30.0)	354	75
	Decreased (10.0–19.9)	25	5
	Increased (30.1–58.9)	95	20
Base excess (*n* = 464) (median = 3.1 mmol/l)	Normal (−2 bis + 2)	127	27
	Decreased (−9.1 bis −2.1)	65	14
	Increased (2.1 bis 28.8)	272	59

## Discussion

In 1954, a study of slaughter cattle found that the incidence of TRP was 80% [1], but when administration of magnets was introduced in the 1960s, the incidence decreased sharply [19]. The results of the present study showed that the yearly incidence of TRP as 7.1%, which was in agreement with the results of other studies in which it ranged from 2 to 12% [2–5]. Traumatic reticuloperitonitis occurred more often in the months of December to April and there was a marked decrease in cases during the summer months, similar to the results of other studies [6, 20], although one study found no association with time of year [21]. There is a lower risk of ingestion of a foreign body during the summer when cattle are grazing than in the winter when they are fed prepared feed. Traumatic reticuloperitonitis is extremely rare in cattle that are kept on pasture year-round [9] because they are more likely to detect foreign bodies in grass than in hay [22]. Contamination of feed with metal foreign bodies is greater with preparation and storage of hay than in fresh forage, although wire is no longer used to tie hay bales. In 33% of the cattle, TRP occurred in the first 2 months postpartum, which was the reproduction stage with the highest number of cases and in agreement with the results of another study (31.3%) [23]. There were no differences among the stages of pregnancy, which was similar to the findings of one study [20] but contrasted the results of one other in which TRP was observed more often in the last trimester of pregnancy [6]. Differentiation of acute and chronic TRP was deliberately omitted for two reasons. Firstly, the definitions of acute and chronic TRP vary considerably depending on which author is cited, and secondly, reliable differentiation is not possible based on history and clinical examination alone. In our experience, cows with chronic TRP are often misdiagnosed as having acute TRP. Reliable differentiation of cattle with acute and chronic TRP can only be determined with a post-mortem examination, which was only carried out in 61 of 503 cattle.

The mean rectal temperature at the time of admission was 39.0 ± 0.7 °C, and only 29% of the cattle had a temperature between 39.1 and 39.5 °C, which is considered typical of TRP. The majority of cattle (52%) had a normal rectal temperature, which was likely attributable to having been ill for several days and treatment before admission to the clinic. In a clinical study of 1446 cattle with acute TRP, the mean rectal temperature was 39.5 °C [20], and in another study of cattle with TRP that had been ill for less than 24 h, it was 39.3 °C [23]. A recently published reference text states that the rectal temperature is 39.5 to 40.0 °C in cattle with acute TRP and within the reference interval or mildly increased in cattle with chronic TRP [9]. A persistent mild increase in rectal temperature is characteristic of chronic inflammation [9].

The mean heart rate was 76 bpm, which was in the upper range of the reference interval and in agreement with the values reported in another study [23] and in a reference text, which states that the mean heart rate in cattle with acute localised TRP is 80 bpm [9]. The mean heart rate in a clinical study of 1446 cattle with acute TRP was slightly higher at 82.4 bpm [20]. A heart rate of more than 90 bpm together with a rectal temperature of more than 40 °C indicates severe complications [9] such as generalised peritonitis or concurrent traumatic pericarditis.

The mean respiratory rate was 28 breaths per minute, which was in the upper range of the reference interval. Constable et al. [9] reported a mean respiratory rate of approximately 30 breaths per minute in cows with TRP. Cattle with TRP do not inhale as deeply as normal in an effort to mitigate pain elicited by movement of the fully-expanded lungs and the diaphragm. An increase in respiratory rate is a compensatory mechanism, which sometimes is misdiagnosed as bronchopneumonia. In cattle with tachypnoea that do not respond to antibiotic therapy, a differential diagnosis should include TRP and other diseases.

Rumen motility is often decreased or absent in cattle with TRP [6, 9]. This was true in the majority (72%) of cattle in the present study and is explained by inhibition of the gastric centre in the medulla oblongata via the vagal nerve because of pain associated with a foreign body [24]. However, decreased rumen motility is a non-specific finding seen in many other diseases of the gastrointestinal tract as well as in systemic disorders. Ruminal tympany, due to decreased eructation because of pain, may be seen in cattle with TRP [6, 8, 9] but occurred in only 51 (10%) of our cases and was recognised as mild bulging of the left paralumbar fossa.

Cattle with TRP often have increased fibre particle length in the faeces [6, 11, 25] because of dysfunction of the sorting mechanisms between the reticulum and omasum, which leads to the movement of incompletely digested feed into the omasum. An increase in fibre particle length occurred in the faeces of 60% of the cattle with TRP in the present study. Fibre particle length is an important indicator of disease of the reticulum, but an increase in length can also be due to dental disease or an increase in the speed of passage of ingesta through the gastrointestinal tract, such as occurs in diarrhoea [17].

Abdominal pain is a cardinal sign of TRP [6, 9–11, 14] and may manifest as arching of the back, grunting or bruxism, which may occur spontaneously or be elicited via foreign body tests. Arching of the back is a sign of parietal pain, and spontaneous grunting is a response to pain caused by reticular contractions. Bruxism is a sign of pain associated with many diseases and is uncommon in cattle with TRP [6]. Signs of pain were seen in 179 (36%) of the cattle in the present study and the most common were bruxism (*n* = 80) and arching of the back (*n* = 68). Spontaneous grunting (*n* = 8) was uncommon; grunting may be difficult to hear, and thus auscultation of the larynx [6] or trachea [26] or placing the palm over the larynx to palpate vibrations [27] is recommended.

The pole test, pinching of the withers and pain percussion were considered by several authors to be the most important part of the clinical examination in cattle suspected of having TRP [6, 28–30]. However, there are few studies that have investigated the response to foreign body testing and the presence of a foreign body in the reticulum [23, 31]. In one study, grunting was elicited by pinching of the withers in 41% and deep palpation with a fist caudal to the sternum in 45% of cows [23], and in another, 16 (61%) of 26 cows had at least one positive foreign body test [30]. In the present study, the pole test was most often positive (43%) followed by pinching of the withers (39%) and pain percussion of the reticular region (24%); at least one test was positive in 58% of cattle, whereas all tests for foreign bodies were negative in 42% of cases. These are sobering results but one must remember that to elicit grunting in cattle with chronic TRP, considerable strength may be required when conducting foreign body tests [6] and in cows with chronic localised peritonitis, the grunt test may be positive, negative or equivocal [9]. Positive swinging and percussion auscultation on the left side was the result of concurrent left displacement of the abomasum in 13 cattle and ruminal atony in another 13. On the right side, positive swinging and percussion auscultation was attributable to intestinal atony or diarrhoea in 82, right displacement of the abomasum in three and caecal dilation in two. The loss of negative pressure in 21 cattle and crepitus in 8 were considered to be signs of peritonitis.

The leukogram and the plasma protein and fibrinogen concentrations are an aid in the diagnosis of TRP in cattle [8]. Acute cases are typically characterised by neutrophilia with a left shift and hyperfibrinogenaemia; however, the leukocyte count may vary in inflammatory disease from severely decreased to severely increased [32]. Leukocyte numbers vary with species and reflect the balance between production and release from the bone marrow and consumption [32]. In contrast to dogs, which have a rapid regenerative capacity and a relatively high bone marrow reserve of neutrophils, cattle have a slow regenerative capacity and a relatively low reserve. Thus dogs with chronic infection usually have persistent neutrophilia, whereas cattle may have normal neutrophil numbers and a normal differential cell count, or even neutropenia with a left shift because of a slow regenerative response. The majority of cattle (54%) in the present study had normal leukocyte counts, which supports the findings of other studies [23, 33–35] in which neutrophilia was not a consistent feature of TRP. Forty-two percent of cattle had leukocytosis with greater than 10,000 leukocytes/μl blood and 90% of these had neutrophilia. Acute localised peritonitis is commonly accompanied by neutrophilia [36, 37], often with a left shift [13, 38]. Leukopenia was rare and only occurred in 4% of cattle in the present study. Leukopenia may occur after the first 1 or 2 days of severe acute inflammation because of migration of circulating neutrophils to the site of inflammation combined with reduced bone marrow response [39]. Stress-related endogenous corticosteroids also can suppress neutrophil numbers. Considering that more than half of a large sample of cows with TRP did not have neutrophilia makes determination of leukocyte numbers a poor diagnostic indicator. However, it is important to note that 272 cattle (54%) had a total leukocyte count within the reference interval (5000–10,000 leukocytes/μl blood) but did not have a differential leukocyte count done to minimise costs. It is not improbable that a left shift or lymphopenia may have been present in some of these animals. Erythropenia is a common sequel of chronic inflammation in cattle [8, 37] and occurred in 4% of the cows in the present study. Chronic illness is the most common cause of mild and clinically insignificant anaemia and the multifactorial pathogenesis of this disorder has been described in detail [40]. Unlike leukocyte numbers, hyperfibrinogenaemia and hyperproteinaemia are good indicators of TRP in cattle and have been linked to this disorder in several studies [41–43]; in fact, in the present study the most important findings of blood analysis were increased fibrinogen concentrations in 69% and increased protein concentrations in 64% of cattle with TRP. Fibrinogen is an acute-phase protein [44] and may be increased as early as 2 to 3 days after the onset of illness [8]. Fibrinogen is often increased in the absence of changes in leukocyte numbers and therefore is the better indicator for inflammation. A recent study on the diagnostic value of different acute-phase proteins concluded that the diagnostic accuracy of fibrinogen was significantly lower than that of serum amyloid A and haptoglobin [43], but other authors favour mainly haptoglobin and fibrinogen as biomarkers for TRP [45, 46] and consider serum amyloid A to be non-specific [46]. High plasma protein concentrations primarily reflect high globulin concentrations and are typical of chronic TRP [41, 47]. However, increases in fibrinogen and plasma protein concentrations are not specific for TRP and the concentrations may be normal or decreased in cows with TRP as seen in this study. The glutaraldehyde clotting test is a point-of-care diagnostic test of considerable practical value used to detect increased gamma globulin and/or fibrinogen concentrations based on polymerisation of fibrinogen and gamma globulins with aldehyde resulting in clot formation of the test sample. The clotting time decreases proportionally with increases in fibrinogen and gamma globulin concentrations. There is a significant correlation between onset of coagulation of the test sample and fibrinogen and gamma globulin concentrations in the blood of cattle. Coagulation time was shorter than 6 min in 75% of tested cattle, and in 49% occurred within 3 min; the respective positive predictive values of these test results for the diagnosis of an inflammatory process are 87.9 and 97.8% [48]. In the latter study, 62.9% of cattle with severe inflammatory changes had clotting times of less than 3 min. However, this test is not specific for TRP and results of blood analysis including the glutaraldehyde test must be interpreted in view of the clinical picture. Reduced clotting time in the glutaraldehyde test, an arched back, fever, reduced rumen motility and positive foreign body tests allow a tentative diagnosis of TRP, but the absence of any of these findings does not rule out TRP. For this reason, the diagnosis should be confirmed by ultrasonographic and radiographic examination of the reticulum. A limitation of the study was that 3.6% of the 503 cattle had concomitant left or right displacement of the abomasum or caecal dilation, which may have affected laboratory findings. Such a low percentage likely had minimal effect on the average and mean values, but is certain to have affected the range of variation. The concomitant disorders would have led to an increase in the maximum values of haematocrit, concentration of urea nitrogen in serum, blood gas variables and concentration of rumen chloride.

## Conclusions

Traumatic reticuloperitonitis cannot be diagnosed on the basis of individual clinical or laboratory criteria because even the most common abnormalities are not seen in all cattle with TRP. Furthermore, many of these findings are not specific for TRP and may occur in other disorders of the abdomen and thorax. A tentative diagnosis may be possible based on all the clinical and laboratory findings.
